# Socio-economic disparities in female genital circumcision: finding from a case-control study in Mahabad, Iran

**DOI:** 10.1186/s12889-022-14247-w

**Published:** 2022-10-07

**Authors:** Shahla Shafaati Laleh, Ghodratollah Roshanaei, Farzaneh Soltani, Fatemeh Ghamari Mehran

**Affiliations:** 1 Student Research Committee, Hamadan University of Medical Sciences, Hamadan, Iran; 2 Department of Biostatics, Hamadan University of Medical Sciences, Hamadan, Iran; 3 Mother and Child Care Research Center, Hamadan University of Medical Sciences, Fahmideh Ave, 6517808836 Hamadan, Iran

**Keywords:** Female genital circumcision, Health status disparities, Socio-economic factors

## Abstract

**Background:**

Female genital circumcision (FGC) is still a challenge in reproductive health. This study investigated socioeconomic disparities in FGC in the Kurdish region of Mahabad, Iran.

**Methods:**

A case-control study was conducted in three comprehensive health centers on 130 circumcised girls as the case group and 130 girls without a history of circumcision as the control group, according to the residential area and the religious sect. The participants completed a previously validated demographic and circumcision information questionnaire. A multivariate logistic regression model with a backward method at a 95% confidence level was used to determine the relationship between socioeconomic variables and FGC.

**Results:**

Multivariate logistic regression showed that a family history of FGC (AOR 9.90; CI 95%: 5.03–19.50), age ranging between 20 and 30 years (AOR 8.55; CI 95%: 3.09–23.62), primary education (AOR 6.6; CI 95%: 1.34–33.22), and mothers with primary education (AOR 5.75; CI 95%: 1.23–26.76) increased the chance of FGC.

**Conclusion:**

The present study provided evidence on socioeconomic factors related to FGC in girls. A family history of FGC, age ranging between 20 and 30 years, and girls’ and their mothers’ education level were strong predictors of FGC. The findings indicate the need to design effective interventions to address these factors to help eradicate FGC.

## Background

The World Health Organization defines female genital circumcision (FGC) as all methods that harm or remove all or a part of female genital organs for non-medical reasons [1]. FGC is mainly performed in Africa and some parts of the Middle East and Asia. However, FGC prevalence and type vary widely among countries [2]. By 2050, approximately one in every three births will occur in 30 countries in Africa and the Middle East, where FGC is widespread [3, 4]. All this is while short-term consequences of FGC including severe pain, bleeding, genital infection, difficulty in urination, and sepsis, as well as its long-term consequences, including chronic pain and infections, menstrual problems, psychological issues, social isolation, and sanitation problems, are well known [5–8]. There are various reasons for FGC, but the main reasons are to be found in strong gender discrimination against women, lack of awareness of the negative consequences of circumcision, and cultural traditions and beliefs. Sexual (controlling or reducing women’s sexual desire), social (girl’s entry into womanhood), health (increasing female fertility), and religious beliefs are commonly associated with FGC [8–10]. FGC is sometimes such a powerful social norm that forces families to circumcise their daughters, even if they are aware of its consequences, in order not to put their marriage at risk [10].

Many studies have examined socioeconomic and demographic factors influencing FGC prevalence [9, 11, 12]. Although place of residence, education level, religion, and ethnicity appear to be associated with FGC prevalence in a given country, the nature of this relationship varies from country to country. For example, in a recent systematic review by El-Dirani et al., a working mother was found to be protective against FGC in one study, a risk factor of FGC in another study, and statistically insignificant in seven studies. Moreover, higher paternal education was protective against FGC in three studies, while two studies showed no association between paternal education and FGC [11]. Significant changes in the religious arrangement of countries, the interaction between religion with place of residence, local customs, and cultural traditions, and even fear of legal consequences or anti-FGC activities can be the main reasons for the lack of continuous relationship between socioeconomic variables and FGC prevalence [12–14].

In Iran, FGC is performed in some southern and western regions [15–17]. It should be noted that FGC is commonly carried out in the eastern part of Kurdistan, Iraq [18], along the border with Kurdistan, Iran. Due to the proximity of these two regions and cultural similarities, FGC is also performed in Kurdistan, Iran. It seems that besides ethnic factors, in cities such as Kurdistan with fewer development indicators, the probability of girls undergoing FGC may be related to some socioeconomic factors. Low literacy level and, consequently, low health knowledge level, and living in deprived areas become an essential obstacle for women and their daughters to achieve full health [19].

According to UNICEF, there has been a significant overall decline in FGC prevalence over the last three decades [20]. In some countries, various steps have been taken, such as restricting and banning this practice and increasing public awareness. Iran’s law does not mention FGC specifically; however, there are no statistics on anti-FGC activities in Iran [21]. Efforts to end the tradition have come primarily from activists who speak to families door-to-door, while officials are silent on the matter. Research on FGC is essential to international, national, and local efforts to end the practice. Given the sensitive nature of the practice, conducting these studies in different regions of Iran can be challenging. Research on FGC in Iran is scarce; hence, there is no reliable data about FGC in the country. The first authoritative study adopting the UNICEF approach has found that FGC is being carried out in at least four major provinces of Iran: West Azerbaijan, Kurdistan, Kermanshah in the west, and Hormozgan in the south [22]. Other studies have also reported varying FGC prevalence rates in different regions. Dehghankhalili et al. (2015), in a cross-sectional study on 780 women in Hormozgan, a southern province of Iran, found that 68.5% of women had undergone FGC [15]. Two other cross-sectional studies in the west of Iran by Pashaei et al. (2012) in Ravansar and Bahrami et al. (2018) in Kamyaran reported FGC prevalence of 55.7% and 50.5%, respectively. However, research, especially case-control studies, on disparities related to FGC is scarce. Identifying socioeconomic factors associated with FGC can help decision-makers and health planners design and implement evidence-based interventions to eradicate FGC. This study investigated socioeconomic disparities that determine FGC in a Kurdish region of Iran.

## Methods

### Study design and participants

The present case-control study was conducted on girls living in Mahabad, a Kurdish region in Iran, in 2018. Mahabad is a green city with a chilly climate in northwest Iran and is about 300km far from the Iran-Iraq border. It has a population of about 133,324 people, and Kurdish is the language predominantly spoken in this region. Muslims living in the Kurdish regions of Iran are mainly “Sunni” in religion and belong to the two sects of Shafi’i and Hanafi.

The study was approved by the Ethics Committee of Hamadan University of Medical Sciences (IR.UMSHA.REC.1397.379), and performed according to the Helsinki Declaration. Informed consent was obtained from all participants, and for girls under 18 years, parent’s or guardian’s consent was obtained prior to the girl’s assent. The participants were assured of the confidentiality of their information. Considering that girls are circumcised at any age, even after puberty and in some cases just before marriage, no age limit was considered in the present study. Accordingly, girls who were not married at the time of sampling and were living in their parents’ house under their support and guardianship were included in the study. Other inclusion criteria were satisfaction with participating in the study, residence in Mahabad City, lack of mental illness, literacy, and ability to understand and speak. Samples not completing more than 10 percent of the questionnaire were excluded from the study.

### Sample size

The G*Power software version 3.1.9.7 was used to calculate the sample size. The G*Power software automatically provides the conventional effect size values suggested by Cohen [23]. In this study, considering the medium effect size (odd = 0.5), type I error (α) = 0.05, and power (1-β) = 0.95, total sample size for each group was calculated to be 130 people.

### Sampling

In the present study, the research environment was comprehensive health centers, and samples were girls referred for routine check-ups, vaccination, or accompanying others. The samples were drawn through cluster sampling across Mahabad City. In this method, each health center was considered a cluster. First, Mahabad City was divided into three geographical regions (north, south, and center), and sampling was carried out according to the number of health centers in these three regions. We reached a total of six clusters, and three clusters (health centers) were randomly selected. Then, 100 eligible subjects were selected from each health center (50 circumcised girls as the case group and 50 uncircumcised girls as the control group). The information of the case group (circumcised girls) was collected consecutively using a non-probability consecutive sampling method until the required number of cases was obtained. The control group consisted of uncircumcised girls matched for the residential area and the religious sect. Data were gathered by trained local interviewers. Informed consent was obtained from each participant, and the socioeconomic and circumcision information questionnaires were completed by self-report in an appropriate room for this purpose in the health centers.

### Study variables

#### Outcome variable

The outcome variable of this study was FGC, defined as cutting and removing all or a part of the clitoris up to the removal of the labia majora. The participants were asked which parts of their genital organs were removed to determine the type of FGC. Since girls are emotional and sensitive, information about the circumcision of girls under 18 was asked from the mother or a reliable companion. It should be noted that all circumcised girls in our study had first-grade genital circumcision, in which the external part of the clitoris is cut.

#### Independent variables

The independent variables were designed by reviewing previous quantitative studies that examined factors associated with FGC or compared factors between women or girls with FGC to those without FGC [11, 12, 24–26]. Associated factors in the individual, parental/household, and community levels were designed as demographic characteristics and circumcision information in a researcher-made questionnaire described below.

#### Study instrument

The researcher-made questionnaire had two sections:

1. Demographic characteristics included age, education level, occupation, number of family members, birth order, parent’s education, parent’s occupation, family income, and housing status.
2. Circumcision information included age at the time of circumcision, circumciser, place of circumcision, a family history of circumcision, knowledge about female circumcision, and source of information.

The questionnaire items were revised and approved by six reproductive health professionals regarding content validity.

### Statistical analysis

Data were analyzed using SPSS/18. The chi-square test was used to assess the relationship between the independent variables and FGC. Variables that showed a significant relationship with FGC at the chi-square level were entered in the univariate and then multivariate regression logistic models with the backward method at a 95% confidence level. In the multivariate model, all demographic and socioeconomic variables were entered into the model. Then, each variable was excluded using the backward method, and the model was finalized in step 9. P-value < 0.05 was considered statistically significant.

## Results

### Demographic characteristics

The majority of participants in the case group were 20–30 years old (63.1%), had academic education (43.8%), and were unemployed (45.4%). The cases’ fathers were self-employed (50%), and their mothers were unemployed (66.9%). Both parents had primary education, with a rate of 50% and 66.9%, respectively. The cases belonged to families of 1–5 people (50.6%), had relatively adequate income (49.2%), and were landlords (81.5%). Also, 77.7% of the participants had a history of FGC in their family.

The majority of the participants in the control group were 20–30 years old (53.8%), had academic education (66.2%), and were students (55.4%). The participants’ fathers were self-employed (47.7%), and their mothers were unemployed (73.8%). Also, the participants’ fathers had secondary education (31.5%), and their mother’s had primary education (38.5%). They belonged to families of 1–5 people (56.2%), had relatively adequate income (48.5%), and were landlords (73.1%). Also, 30% of the controls had a history of FGC in their family (Table1).

**Table 1:** Comparison of demographic and socio-economic factors in term of two groups (N = 260)

Variables		Without FGC N (%)	FGC N (%)	OR	CI, 95%	P -value*
Age(year)	20 >	33(25.4)	37(28.5)	2.75	1.18, 6.39	0.019
	20–30	70(53.8)	82(63.1)	2.87	1.33,6.21	0.007
	30<	27(20.8)	11(20.8)	ref		
Education	Elementary	4(3.1)	14(10.8)	5.28	1.65, 16.85	0.005
	Guidance	20(15.4)	27(20.8)	2.03	1.04, 3.97	0.037
	Diploma	20(15.4)	32(24.6)	2.41	1.25, 4.69	0.008
	Academic	86(66.2)	57(43.8)	ref		
Occupation status	Employed	19(14.6)	26(20.0)	0.90	0.44, 1.85	0.784
	Student	72(55.4)	45(34.6)	0.41	0.23, 0.71	0.002
	Unemployed	39(30.0)	59(45.4)	ref		
Father’s job	Government’s employee	45(34.6)	32(25.0)	0.71	0.21, 2.40	0.584
	worker	17(13.1)	20(15.6)	1.12	0.34, 3.68	0.841
	Self-employed	62(47.7)	70(54.7)	1.17	0.32,4.33	0.807
	Unemployed	6(4.7)	6(4.7)	ref		
Father’s education	Elementary	28(21.5)	64(50.0)	5.48	2.31, 12.97	0.001
	Guidance	41(31.5)	26(20.3)	1.52	0.62, 3.69	0.353
	Diploma	37(28.5)	28(21.9)	1.81	0.74, 4.40	0.187
	Academic	24(18.5)	10(7.8)	ref		
Mother’s job	Employed	34(26.2)	19(14.6)	0.48	0.25, 0.90	0.022
	Unemployed	96(73.8)	111(85.4)	ref		
Mother’s education	Elementary	50(38.5)	87(66.9)	6.96	1.87, 25.84	0.004
	Guidance	48(36.9)	28(20.0)	2.16	0.56, 8.37	0.262
	Diploma	20(15.4)	14(10.8)	2.80	0.66, 11.79	0.160
	Academic	12(9.2)	3(2.3)	ref		
Family members size	1–5	73(56.2)	66(50.8)	0.67	0.14, 3.14	0.620
	6–10	54(41.5)	60(46.2)	0.83	0.17, 3.89	0.817
	11≥	3(2.3)	4(3.1)	ref		
Monthly family income	Less than adequate	15(11.5)	8(6.2)	0.38	0.09, 1.59	0.187
	relatively adequate	63(48.5)	64(49.2)	0.72	0.21, 2.40	0.600
	adequate	47(36.2)	51(39.2)	0.77	0.23, 2.61	0.681
	More than adequate	5(3.8)	7(5.4)	ref		
housing status	Rental	28(21.5)	20(15.4)	1.25	0.32, 4.85	0.747
	Personal residence	95(73.1)	106(81.5)	1.95	0.55, 6.87	0.298
	Residence with family	7(5.4)	4(3.1)	ref		
Family history of FGC	Yes	39(30.0)	101(77.7)	8.12	4.65, 14.19	0.001
	No	91(70.0)	29(22.3)	ref		

FGC: Female Genital Circumcision; OR: Odds Ratio; CI: Confidence Interval

*Univariate logistic regression model

### FGC related characteristics

45% of the girls had been circumcised under five years, and about half of them were the first child in the family. Also, circumcision was performed on more than half of the girls by local women, and more than 90% of them were circumcised at home. 60% of the circumcised girls had information about circumcision, with the Internet being their most common source of information. Most of the girls did not have pain in their circumcision area (Table2).

**Table 2:** Descriptive characteristics of circumcised girls (n = 130)

Variables		Frequency	%
Age of circumcision (yr.)	1–4	59	45.4
	5–9	52	40
	10≤	19	14.6
Birth order	First	50	38.5
	Second	32	24.6
	Third	21	16.2
	Fourth≥	27	20.8
Circumciser	Rural women	73	65.2
	Rural midwife	18	13.8
	Educated midwife	7	5.4
	Relatives	5	3.8
	Others	27	20.8
Place of circumcision	Home	122	93.8
	Hospital	4	3.1
	Health center	4	3.1
Having information about FGC	Yes	78	60.00
	No	52	40.00
Source of information about FGC	Book	26	20
	Internet	31	23.8
	Mother	9	6.9
	Sister	6	4.6
	Friends	5	3.8
	Doctors	14	10.8
	No response	39	30
Pain in scars	Yes	14	10.8
	No	116	86.2

#### Socio-economic predictive factors of FGC

The relationship between each demographic and socioeconomic variable with the circumcision status was investigated using the univariate regression model. The results showed that the variables of age, education, occupation, parents’ education, mother’s occupation, and previous history of circumcision in their family were related to an increase in the chance of circumcision. Accordingly, the chance of circumcision was 2.87 [CI 95%; 1.33–6.21] times higher in girls aged 20 to 30 than in those over 30. This chance was 5.28 [CI95%; 1.65–16.85] times higher in girls with primary education than in those with academic education. The probability of circumcision was 0.41 [CI 95%; 1.25–4.69] times more in students than in unemployed women. The chance of circumcision was also 6.96 [CI 95%; 1.87–25.84] and 0.48 [CI 95%; 0.25–0.95] times higher in girls circumcised with mothers with primary education and employed mothers than in girls with academic education and unemployed mothers, respectively. Furthermore, this probability in participants whose fathers had primary education was 5.48 [CI 95%; 2.31–12.97] times. A family history of circumcision increased the odds of female circumcision by 8.12 [CI 95%; 4.65–14.19] times (Table2).

The participants were adjusted regarding occupation, father’s job, father’s education, mother’s job, number of family members, monthly family income, and housing status. Multivariate logistic analysis using the backward method showed that age, education level, mother’s education, and the family history of circumcision were the predictive variables of circumcision. Accordingly, the chance of circumcision was respectively 5.77 [CI 95%; 1.86–17.84] and 8.55 [CI 95%; 3.09–23.62] times higher in girls under 20 and in the age group of 20 to 30 years than in those over 30 years. This change was 6.6 [CI 95%; 1.34–33.22] times higher in girls with primary education than in those with academic education. Also, the chance of circumcision was 5.75 [CI 95%; 1.23–26.76] times higher in girls with mothers with primary education than in girls with mothers with academic education. Also, the family history of circumcision increased the probability of female circumcision by 9.90 [CI 95%: 5.03–19.50] times (Table3).

**Table 3:** The socioeconomic - related disparities in female genital circumcision

Variables		OR	CI, 95%	P-value*
Age	20>	5.77	1.86,17.84	0.002
	20–30	8.55	3.09,23.62	0.001
	30<	ref		
Education	Elementary	6.68	1.34,33.22	0.020
	Guidance	2.25	0.80,6.29	0.120
	Diploma	2.20	0.95,5.09	0.065
	Academic	ref		
Mother’s education	Elementary	5.75	1.23,26.76	0.026
	Guidance	1.41	0.29,6.79	0.665
	Diploma	2.35	0.44,12.64	0.316
	Academic	ref		
Family history of FGC	Yes	9.90	5.03,19.50	0.001
	No	ref		

* Multivariate Logistic analysis backward method (Occupation, Father’s job, Father’s education, Mother’s job, Household members, Family income, housing status variable(s) removed on the step 9)

#### Circumcision performance related factors

The socioeconomic-related factors, including age at the time of FGC, circumciser, and place of circumcision, were examined. The results showed that age at the time of circumcision was significantly related to the family’s housing status. Thus, circumcision was more prevalent in girls under five years in families who were landlords than in other groups (P = 0.034). Circumcision by local women was also significantly higher in those whose fathers had primary education (P = 0.003) and whose family income was relatively adequate (P = 0.011). The place of the circumcision was significantly related to the father’s occupation and education status. Thus, girls whose fathers had primary education (P = 0.034) and were retired (P = P = 0.024) were more likely to be circumcised at home. The mother’s occupation and education level had no significant relationship with the three circumcision performance-related variables (P > 0/05).

## Discussion

About 200million women suffer from mutilation in 30 countries, and more girls are being exposed to circumcision per year due to the increasing rate of population in such countries [2]. According to the results of the present study, a family history of circumcision increased the probability of girls being circumcised by almost ten times. This suggests that FGC is passed from generation to generation within families. A family history of circumcision has had a strong relationship with female circumcision in Kurdistan, Iraq [27]. Also, the systematic review of El-Dirani et al. showed that this variable was a risk factor for FGC in most of the studies [11].

The majority of circumcised girls in our study (45.4%) were circumcised under age five. FGC is usually performed between ages 8 and 14. However, female circumcision cannot be restricted to a specific age, and girls are constantly exposed to circumcision from birth to puberty. EDHS 2008 stated that all female circumcisions were performed before age 15. While the age of circumcision varies between tribes and countries, it seems that Muslims circumcise their daughters at a younger age [28]. Using data for 7,620 women interviewed in the 2003 NDHS, Kandala et al. found that the rate of female circumcision at about five years of age is similar in both urban and rural areas, but circumcision over five years of age seems to be more common in urban areas [29]. A cross-sectional study on 492 people in three refugee camps in Somalia showed that circumcision prevalence increased with age, with 52% and 95% of girls being circumcised at 7–8 and 11–12 years of age, respectively [30]. In our study, FGC was performed by rural women in more than half of the cases and at home in more than 90% of the cases. Consistent with our findings, Pashaei et al., in a cross-sectional study in Ravansar (Iran), found that 96.4% of girls were circumcised by local women at home [15]. Numerous studies have shown that FGC is usually performed by indigenous people with rudimentary tools [31, 32]. However, in some countries, such as Sudan and Egypt, trained midwives and physicians have replaced traditional circumcisers as a way of harm reduction [33, 34]. Al Awar et al., in their cross-sectional study conducted in the United Arab Emirates, reported that 73.7% of circumcision in girls was performed by health professionals/at private clinics [32]. However, the involvement of medical specialists in female circumcision can help prevent the spread of infectious diseases by preventing the use of non-sterile instruments and unnecessary FGC procedures.

In the present study, girls with primary education were circumcised nearly seven times more than those with academic education. The lack of formal education was significantly associated with an increased risk of FGC in several studies [30, 31, 35]. The Egyptian Demographic Health Survey 2008 (EDHS) showed that the probability of circumcision decreased with increasing education level and was higher among women belonging to lower social classes [28]. Also, in a study conducted by Gebremariam et al. (2016) on 679 young female students from Somali, participants’ educational level was a significant independent predictor of circumcision [14]. Other studies have shown that education and self-empowerment are associated with circumcision rejection [36–39]. For example, a community-based cross-sectional study conducted in Ethiopia showed that uncircumcised women examined in high school or at higher levels did not want to continue circumcision compared to those who were illiterate [40]. In general, education can be interpreted as a form of women’s empowerment that can equalize the balance between them and men [41]. Since circumcision is performed before formal education begins, many believe that the mother’s education level is a more significant variable for assessing the relationship between FGC and education [19]. Mothers’ education is an essential predictor of FGC in young girls, as several studies have reported that as women’s education increases, the incidence of female circumcision decreases and vice versa [19]. In a cross-sectional study by Ali et al. on 3353 young women in Egypt, girls with illiterate parents reported significantly higher rates of circumcision [42]. Gajaa et al. (2014), in a cross-sectional study, determined associated factors of circumcision among daughters in Nigeria. They found that daughters of uneducated mothers were 3.5 times more likely to be circumcised than daughters of educated mothers [43].

Another noteworthy point in the present study is that fathers’ education and occupation, as well as family income, were correlated with the circumciser and the place of circumcision. Thus, girls whose fathers had primary education and were retired and whose family incomes were lower were mostly circumcised by a rural midwife at home. It seems that while low-educated mothers are more likely to circumcise their daughters, low-educated fathers and the low socioeconomic status of the family affect how circumcision is performed. These results show the impact of the socioeconomic level of families on the quantity and quality of female circumcision. Our results highlighted the importance of modifiable variables, such as girls’ and parents’ education (primarily mothers’ education), as predictors of FGC. It is assumed that educated women are primarily aware of their rights and are more capable of demanding and defending these rights for themselves and their daughters. Also, education may reduce women’s dependence and give them greater economic stability.

The main limitation of this study is that the participants were only Kurdish girls living in a Kurdish region in Iran, which impedes the generalization of the study results to other ethnic groups and nationalities. Another limitation of this study is the use of a self-reported questionnaire that increases the chance of recall and social desirability bias. The inclusion criterion of “literacy” may have excluded girls at higher risk of circumcision. In addition, a wide confidence interval is noted in some variables due to the small sample size. Further studies with a larger sample size from different settings are needed to increase the accuracy and generalizability of the findings obtained from this study.

## Conclusion

The present study has provided evidence on socioeconomic factors associated with FGC in a Kurdish region of Iran and can help design effective interventions to eradicate this harmful practice. A family history of FGC, age of 20–30 years, and education level of girls and their mothers were found as predictors of FGC. Considering these strong predictive factors, we recommend targeted interventions based on addressing the modifiable variable. Accordingly, empowering women by creating education opportunities can lead to the success of global efforts to fight and eradicate FGC.

## Data Availability

The datasets used and/or analyzed for this study are available by the corresponding author upon reasonable request.

## Declarations

was approved by the ethics committee of the Research Council of Hamadan University of Medical Sciences (IR.UMSHA.REC.1397.379). Written consent was obtained from the participants, and they were assured of the confidentiality of their information.

## Abbreviations

FGC: Female Genital Circumcision
WHO: World Health Organization
UNICEF: United Nations International Children’s Emergency Fund
